# The Effect of Ginsenoside RB1, Diazoxide, and 5-Hydroxydecanoate on Hypoxia-Reoxygenation Injury of H9C2 Cardiomyocytes

**DOI:** 10.1155/2019/6046405

**Published:** 2019-12-13

**Authors:** Heng Zhang, Xiao Wang, Yihua Ma, Yueping Shi

**Affiliations:** First Affiliated Hospital of Jinzhou Medical University, Jinzhou, Liaoning 121001, China

## Abstract

This study was aimed to investigate whether ginsenoside Rb1 (GS-Rb1) from the cardioprotective Chinese medicine ginseng can reduce hypoxia-reoxygenation (HR)-induced damage to cardiomyocytes by protecting the mitochondria. Mitochondria-mediated apoptosis plays a key role during myocardial ischemia-reperfusion injury (MIRI). When MIRI occurs, the continuous opening of the mitochondrial permeability transition pore (mPTP) causes mitochondrial damage and ultimately leads to apoptosis. We treated H9c2 cells, derived from rat embryonic cardiomyoblasts, with GS-Rb1, diazoxide, and 5-hydroxydecanoate (5-HD), using HR to simulate MIRI. We found that GS-Rb1 can reduce mPTP by stabilizing the mitochondrial membrane potential (MMP) and by reducing reactive oxygen species (ROS) during HR. This protects the mitochondria by reducing the release of cytochrome c and the expression of cleaved-caspase-3 in the cytoplasm, ultimately reducing apoptosis. During this process, GS-Rb1 and diazoxide showed similar effects. These findings provide some evidence for a protective effect of GS-Rb1 treatment on MIRI.

## 1. Introduction

Myocardial ischemia-reperfusion injury (MIRI) refers to the pathological process of progressive aggravation of the ischemic myocardium that often occurs during various reperfusion treatments after myocardial infarction. Since MIRI seriously reduces the effect of reperfusion therapy, its prevention and treatment is critical. The mechanism of MIRI is very complex and includes myocardial apoptosis [1]. Mitochondria are among the most important organelles in eukaryotic cells. The stable structure and function of the mitochondria are prerequisites for the occurrence of their normal activities, such as electron transport and the tricarboxylic acid cycle. In addition to the synthesis of ATP, mitochondria also play an important role in promoting cell proliferation, signal transmission, and apoptosis, the latter centered on the activation of the cysteinyl aspartate-specific proteinase (caspase) family [2]. Mitochondria occupy 30% of the volume of cardiomyocytes and produce approximately 30 kg of ATP per day to maintain heart contractions [3]. Therefore, damage to mitochondria may be an important cause of ischemia-reperfusion injury in cardiomyocytes.

**Figure 1:**
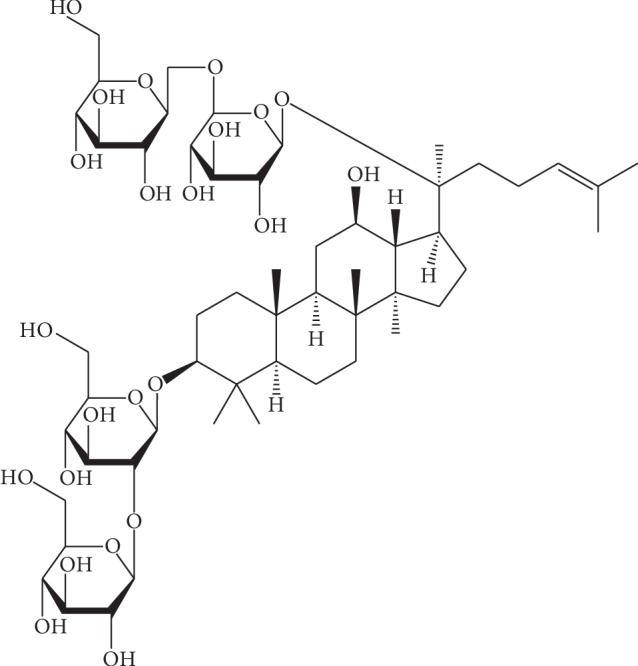
Chemical structure of ginsenoside Rb1.

Ginseng is a traditional Chinese medicine that is widely used to treat ischemic heart disease. GS-Rb1, one of the main components of ginseng, is a triterpenoid saponin compound (Figure 1) that has significant anticancer effects as well as protective effects on nerve cells and cardiomyocytes [4–7]. Here, we pretreated H9c2 cardiomyocytes with GS-Rb1, followed by simulation of MIRI by HR and explored the effect of GS-Rb1 on H9c2 cells during HR and whether this effect is related to mitochondria were explored.

Diazoxide is a specific agonist of the mitochondrial ATP-sensitive potassium (MitoK_ATP_) channel, which has been shown to protect cardiomyocyte mitochondria during MIRI [8]. In this study, diazoxide was added as a positive control. 5-HD, as a specific blocker of MitoK_ATP_, was coincubated together with GS-Rb1 to observe if the effect of GS-Rb1 was affected by 5-HD.

## 2. Materials and Methods

### 2.1. Cell Culture

The H9c2 cell line derived from rat embryonic cardiomyoblasts was purchased from Bosterbio (Wuhan, China). Cells were seeded into cell culture dishes and cultured in DMEM/F12 (Sigma-Aldrich, USA) supplemented with 10% fetal bovine serum (Clark Bioscience, USA) and 1% penicillin/streptomycin (Solarbio, Beijing, China). The culture was maintained in an incubator (Sanyo, Japan) containing 5% CO_2_ at 37°C.

### 2.2. Analysis of the Cytotoxicity of GS-Rb1 on H9c2 Cells

Cytotoxicity was assessed using a Cell Counting Kit-8 (CCK8; Beyotime, Shanghai, China) [9]. GS-Rb1 (≥98%, Solarbio, Beijing, China) was dissolved in serum-free DMEM/F12 and filtered through a 0.22 *μ*m filter. H9c2 cells were seeded into 96-well plates at a density of 1 × 10^4^ per well, and GS-Rb1 was added after 12 hours of culture at final concentrations of 50 *μ*M, 100 *μ*M, 150 *μ*M, and 200 *μ*M, respectively. Controls received serum-free DMEM/F12 without GS-Rb1. After 6 h, 12 h, or 18 h, the medium was removed, and 10 *μ*l CCK-8 solution and 100 *μ*l medium were added to each well for 1 hour. Next, the OD value at 450 nm of each well was read by a multifunction microplate reader (TECAN, Switzerland). Blank values from wells containing only DMEM/F12 without cells were subtracted. The cell viability of the 0 *μ*M group was set at 100%, and the relative cell viability of the other groups was calculated. OD values were determined for three replicate wells for each experimental point.

### 2.3. Hypoxia-Reoxygenation (HR)

Cells were grown to approximately 80% confluence. Growth medium was withdrawn, the cells were washed with phosphate-buffered saline (PBS), and glucose-free DMEM (Solarbio, Beijing, China) without serum was added. The AnaeroPack system was used to construct an anaerobic environment according to the method of Takahashi et al. [10]. Briefly, the cells were placed together with an opened AnaeroPack (Mitsubishi, Japan) in a sealed airtight culture bag and cultured at 37°C. The oxygen level in the bag was measured by using an oxygen indicator (Haibo, China), which turned from blue to pink in an oxygen-free environment. The AnaeroPack creates an anaerobic environment by absorbing oxygen and releasing carbon dioxide; it generally reduces the atmospheric oxygen content to less than 1% within 1 hour and increases carbon dioxide levels to 5% [11]. The airtight culture bag was opened after 3 hours of hypoxia; the cells were quickly removed from the bag, and the glucose-free DMEM was replaced with serum-free DMEM/F12. Then, the cells were placed in a regular 5% CO_2_ incubator for 3 hours of reoxygenation at 37°C.

### 2.4. ATP Content Assay

H9c2 cells were seeded at a density of 2 × 10^3^ cells/well into opaque black 96-well plates (Corning, USA) and divided into five groups, A–E, with 3 replicate wells per group. Cells in Group A were not subjected to HR and were seeded in separate plate. After 12 hours of cell culture, cells from group C were incubated with 100 *μ*M GS-Rb1 for 18 h. Cells from group D were supplemented with 500 *μ*M 5-HD for 10 min; then, 100 *μ*M of GS-Rb1 was added and the cells were incubated for 18 h (500 *μ*M 5-HD alone had no impact on cell vitality [12]). Cells from group E were treated with 100 *μ*M diazoxide for 10 min before hypoxia was induced. Hypoxia began after all pretreatments were completed; the culture medium of the B, C, D, and E groups was removed, the cells were washed twice with PBS, and then glucose-free medium without serum was added. The plates were placed in an airtight culture bag with an AnaeroPack and an oxygen indicator. After 3 hours of hypoxia, plates were removed from the airtight culture bag and the glucose-free medium was replaced with serum-free DMEM/F12 and placed in an incubator containing 5% CO_2_ at 37°C. After 3 hours of reoxygenation, the culture medium of every well was removed, 100 *μ*l serum-free DMEM/F12 and 100 *μ*l CellTiter-LumiTM luminescence assay reagent were added to each well, and the mixture was shaken for 2 min and incubated for 10 min at room temperature [13]. The chemiluminescence of each well was measured by a microplate reader, and the relative ATP content of groups B–E was calculated by considering the ATP content of group A as 100%. CellTiter-LumiTM luminescence assay reagents were purchased from Beyotime (Shanghai, China), and 5-HD (≥97%) and diazoxide (100%) were purchased from Sigma-Aldrich (USA). The concentrations of 5-HD and diazoxide are according to the study of Murata et al. [14].

### 2.5. Lactate Dehydrogenase (LDH) Release Assay

The H9c2 cells were seeded at a density of 1 × 10^5^ cells/well into a 24-well plate, and 1 ml of the medium was added to each well. The cells were subjected to HR as described above. The culture medium of each group was collected at the end of the hypoxia condition, and at the end of the reoxygenation period and after centrifugation, the LDH contents were determined using an LDH assay kit (Wanleibio, Shenyang, China). The OD value at 450 nm was read by a microplate reader, and the LDH content was calculated according to the formula provided in the instruction manual.

### 2.6. Mitochondrial Membrane Potential (MMP) Assay

Rhodamine 123 (Rh123) is a lipophilic cationic fluorescent dye that is very sensitive to MMP and depends on the MMP to enter mitochondria. When MMP is high, Rh123 aggregates in the mitochondrial matrix and undergoes fluorescence quenching, which reduces the fluorescence intensity of the cells; when the MMP is low, the Rh123 that is distributed in the cells cannot aggregate in the mitochondria and emits strong green fluorescence [15, 16]. Rh123 was dissolved in DMSO (1 mg/ml), filtered through a 0.22 *μ*m organic filter, and diluted in serum-free DMEM/F12. H9c2 cells were seeded at a density of 1 × 10^5^ cells per well into 24-well plates, 1 ml of medium was added to each well, and the cells were subjected to HR as described above. The medium was removed after HR, cells were washed once with PBS, Rh123 solution was added to every well, at a final concentration of 1 *μ*M, and the cells were incubated at 37°C for 15 min in the dark. The Rh123-containing medium was removed, the cells were washed twice with PBS, and the fluorescence intensity of each group was observed by a fluorescence microscope (Olympus, Japan). Fluorescence intensity was negatively correlated with MMP; therefore, we used the reciprocal of the average fluorescence intensity (AREA/IOD) to represent MMP. The average fluorescence intensity was obtained from five fields of view. The MMP of the control cells was regarded as 100%, and the relative MMP of treated cells was calculated accordingly. The Rh123 (≥85%) was purchased from Sigma-Aldrich (USA).

### 2.7. Assessment of Reactive Oxygen Species (ROS) by DCFH-DA

DCFH-DA is a commonly used reactive oxygen species (ROS) fluorescent probe; when DCFH-DA enters cells, it can be hydrolyzed into DCFH by esterase and oxidized by ROS to DCF, emitting green fluorescence [17]. The DCFH-DA solution (1 mM) was purchased from Wanleibio (Shenyang, China). The H9c2 cells were seeded into a 24-well plate at a density of 1 × 10^5^ cells/well, 1 ml of the medium was added to each well, and the cells were subjected to HR as described above. The medium was removed following HR, and the cells were washed once with PBS. DCFH-DA was added into every well at a final concentration of 10 *μ*M in serum-free DMEM/F12, and the cells were incubated for 30 min at 37°C in the dark. Subsequently, the cells were washed twice with PBS, and the fluorescence intensity was observed by fluorescence microscopy. The fluorescence intensity was positively correlated with the amount of ROS produced in the cells; thus, we used the average fluorescence intensity (IOD/AREA) to represent the intracellular ROS content, and the average intensity was obtained from five high powered fields.

### 2.8. Opening of the Mitochondrial Permeability Transition Pore (mPTP)

To assay the opening of the mPTP, we employed the method of Petronilli et al. [18] based on coincubation of the nonfluorescent acetomethoxy derivate of calcein (calcein-AM) and CoCl_2_. Calcein-AM was dissolved in DMSO at 1 mg/ml, filtered through a 0.22 *μ*m organic filter, and diluted in serum-free DMEM/F12. CoCl_2_ was dissolved and diluted in serum-free DMEM/F12. The H9c2 cells were seeded at a density of 1 × 10^5^ per well into a 24-well plate in 1 ml of medium, and the cells were subjected to HR as described above. Following HR, the medium was removed, the cells were washed once with PBS, and calcein-AM and CoCl_2_ was added into each well. The final concentration of calcein-AM was 1 *μ*M and that of CoCl_2_ was 2 mM; the cells were incubated for 15 min at 37°C in the dark. Next, the medium was withdrawn and the cells were washed twice with PBS. The fluorescence intensity of each group was observed by a fluorescence microscope. Calcein-AM is a hydrophobic compound that easily enters cells and mitochondria and upon hydrolysis to calcein by esterase emits green fluorescence intracellularly. CoCl_2_ can enter the cell to quench the fluorescence of calcein, but it cannot enter the mitochondria; only when mPTP is opened can CoCl_2_ enter the mitochondria to quench the fluorescence of calcein. Therefore, when calcein-AM is coincubated with CoCl_2_, the fluorescence intensity is negatively correlated with the opening of mPTP. Thus, we used the reciprocal of the average fluorescence intensity (AREA/IOD) to represent the opening of mPTP. The average fluorescence intensity of five fields of view was obtained for each group. Calcein-AM (≥96%) and CoCl_2_ (99.7%) were purchased from Aladin (Shanghai, China).

### 2.9. Apoptosis Rate Assay

To improve accuracy, we evaluated the apoptosis rate using both Hoechst 33342 staining [19] and TUNEL/DAPI staining [20]. H9c2 cells were seeded at a density of 1 × 10^5^ cells/well into 24-well plates; 1 ml of medium was added to each well, and the cells were subjected to HR as described above. The medium was removed after HR, and the cells were washed once with PBS.

#### 2.9.1. Hoechst 33342 Staining

A 200 *μ*l Hoechst 33342 solution (Wanleibio, Shenyang, China) was added to each well and incubated for 30 min at 37°C in the dark. Cells were washed twice with PBS after removing the Hoechst 33342, and the number of apoptotic cells in each group was counted by fluorescence microscopy. Excited by ultraviolet light, the normal nucleus has normal morphology with low-intensity blue fluorescence, while the apoptotic nucleus has abnormal morphology, such as fragmentation and aggregation, with high-intensity fluorescence. The apoptosis rate is the ratio of apoptotic nuclei to normal nuclei. The average ratio from five fields from each group was obtained.

#### 2.9.2. TUNEL/DAPI Staining

The cells were fixed with precooled 4% paraformaldehyde solution for 30 minutes, washed once with PBS, lysed for 10 min with enhanced immunostaining permeabilization buffer, washed twice with PBS, and then 5 *μ*l of terminal deoxyribonucleotidyl transferase (TDT) enzyme and 45 *μ*l of FITC labeling solution were added, and covered with antievaporation membrane followed by incubation at 37°C for 1 h in the dark; then, the cells were washed three times with PBS. Next, 200 *μ*l of antifade mounting medium with DAPI was added to every well, and the number of apoptotic cells in each group was counted by fluorescence microscopy. Excited by blue-green light, green apoptotic nuclei can be observed, and excited by ultraviolet light, all nuclei are blue. The ratio of green nuclei to blue nuclei is the apoptosis rate. The average apoptosis rate was obtained by observing five fields from each group. One Step TUNEL Apoptosis Assay Kit, 4% Paraformaldehyde Fix Solution, Enhanced Immunostaining Permeabilization Buffer, and Antifade Mounting Medium with DAPI were purchased from Beyotime (Shanghai, China).

### 2.10. Apoptosis-Related Protein Expression

The H9c2 cells were seeded into 100-mm cell culture dishes and subjected to HR as described above. After reoxygenation, the medium was collected and the cells were digested with 0.25% trypsin. The cells were resuspended in the collected medium and centrifuged. A cell mitochondria isolation kit was used to separate cytoplasmic proteins from the mitochondria, and the mitochondria were lysed using mitochondrial lysis solution [21]. The cytoplasmic proteins and mitochondrial proteins were quantified using a BCA protein assay kit. The protein samples were added to a loading buffer with SDS and then heated at 100°C for 5 minutes. After cooling, aliquots of the mixture were frozen at −20°C until use. Proteins were separated on a 15% sodium dodecyl sulfate polyacrylamide gel (SDS-PAGE) with 20 *μ*g of total protein added to each well. After electrophoresis in Tris-glycine buffer, the proteins were transferred to a 0.22 *μ*m PVDF membrane. The membrane was blocked with 5% nonfat milk powder in Tris-buffered saline containing 0.1% Tween-20 (TBST) at room temperature for 2 hours and then incubated at 4°C overnight with the primary antibodies. After washing with TBST, the membrane was incubated with the secondary antibody for 1 hour at room temperature. After washing with TBST, ECL chemiluminescence reagent was added to the PVDF membrane, and a chemiluminescence imaging system (Bio-Rad, USA) was used for exposure imaging. The cell mitochondria isolation kit and the ECL chemiluminescence kit were purchased from Beyotime (Shanghai, China); primary antibodies (caspase-3/cleaved-caspase-3, cytochrome C, *β*-tubulin, VDAC1), secondary antibodies (HRP-labeled goat anti-rabbit IgG), and the BCA protein assay kit were purchased from Wanleibio (Shenyang, China). Antibodies were diluted following the manufacturer's instructions.

### 2.11. Statistical Analysis

All experiments were repeated three times. The data were statistically analysed by SPSS 17.0 and expressed as mean ± SEM. Statistical comparison was carried out with one-way ANOVA. *p* < 0.05 was considered statistically significant (^*∗*^*p* < 0.05, ^*∗∗*^*p* < 0.01).

## 3. Results

### 3.1. Pretreatment with GS-Rb1 Does Not Affect the Viability of H9c2 Cells

**Figure 2:**
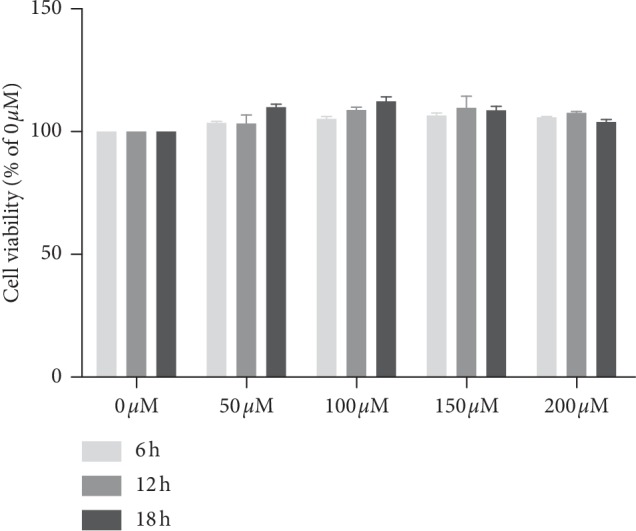
Ginsenoside Rb1 (GS- Rb1) is not cytotoxic to H9c2 cells at concentrations of up to 200 *μ*M and incubation times up to 18 h. GS-Rb1 appeared to enhance the viability of H9c2 cells, and this effect became more obvious with the prolongation of treatment time. Cell viability in the absence of GS-Rb1 (0 *μ*M) was set at 100%.

The viability of H9c2 cells was analysed in the absence or presence of various concentrations of GS-Rb1. The cell viability in the absence of GS-Rb1 was regarded as 100%. No cytotoxicity was observed in the H9c2 cells that were treated with different concentrations of GS-Rb1 for 6 hours, 12 hours, or 18 hours, and GS-Rb-1 actually appeared to moderately enhance the growth of H9c2 cells. The maximal effect was reached at 100 *μ*M and became more obvious with the prolonged treatment time (Figure 2). The subsequent research was carried out under the conditions of 18 h and 100 *μ*M GS-Rb-1. Under these conditions, the viability of H9c2 cells was optimal.

### 3.2. GS-Rb1 Protects Mitochondrial Function during HR

**Figure 3:**
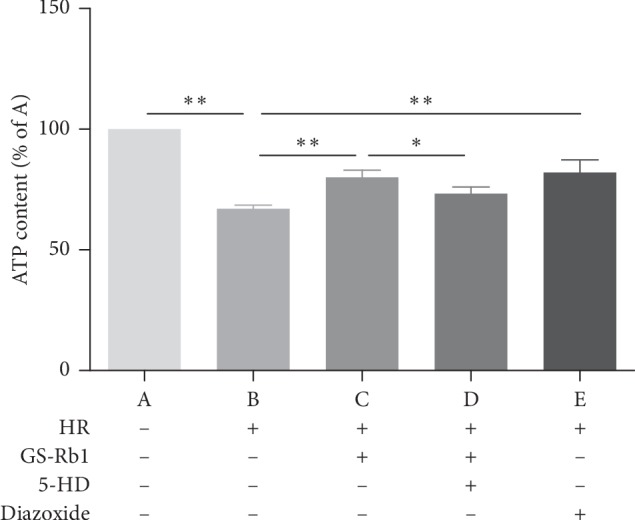
Mitochondrial function and cell viability assessed by ATP content assay. The content of ATP in H9c2 cells decreased during HR compared with control cells (B vs. A ^*∗∗*^*p* < 0.01), and pretreatment with GS-Rb1 (100 *μ*M) can significantly reduce the decrease in ATP content in H9c2 cells during HR compared with nontreated cells (C vs. B ^*∗∗*^*p* < 0.01), similar to the diazoxide (E vs. B ^*∗∗*^*p* < 0.01), and this effect can be attenuated by the 5-HD (500 *μ*M) (D vs. C ^*∗*^*p* < 0.05; D vs. B ^*∗*^*p* < 0.05). HR, hypoxia-reoxygenation; GS-Rb1, ginsenoside Rb1; 5-HD, 5-hydroxydecanoate.

ATP levels can be used as an indicator of mitochondrial function and cell viability. The ATP content of the control group (H9c2 cells not subjected to HR and grown in the absence of GS-Rb-1) was set at 100%. The ATP content decreased significantly in cells subjected to HR in the absence of GS-RB-1 compared with the control (Figure 3, column B vs. A, ^*∗∗*^*p* < 0.01) indicating decreased mitochondrial function. The ATP content of the GS-Rb1 pretreatment group was significantly higher than that of the HR group in the absence of GS-Rb1 (Figure 3, column C vs. B, ^*∗∗*^*p* < 0.01) indicating a protective effect of GS-Rb1 addition, and this protective effect could in turn be attenuated by the addition of the 5-HD (Figure 3, column D vs. C, ^*∗*^*p* < 0.05, column D vs. B, ^*∗*^*p* < 0.05). Diazoxide produced a similar protective effect as GS-Rb1 during HR, and the ATP level was significantly higher than that of the HR group without GS-Rb1 or diazoxide (Figure 3, column E vs. B, ^*∗∗*^*p* < 0.01). Thus, GS-Rb1 can protect the mitochondrial function of H9c2 cells and increase cell viability during HR, and this protection can be partially eliminated by the 5-HD.

### 3.3. GS-Rb1 Reduces the Release of LDH during HR

**Figure 4:**
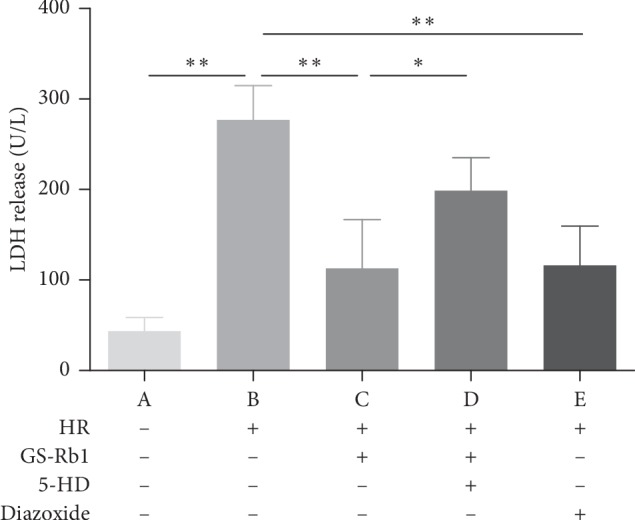
Protective effect of GS-Rb1 on the level of LDH release is related to the extent of damage to H9c2 cells. The release of LDH from H9c2 cells increased during HR compared with cells under normal conditions (B vs. A ^*∗∗*^*p* < 0.01). Pretreatment with GS-Rb1 significantly reduced the release of LDH from H9c2 cells during HR compared with nontreated cells (C vs. B, ^*∗∗*^*p* < 0.01), similar to the effect of diazoxide (E vs. B ^*∗∗*^*p* < 0.01), and this effect can be partially eliminated by pretreatment with 5-HD (C vs. D ^*∗*^*p* < 0.05). LDH, lactase dehydrogenase; HR, hypoxia-reoxygenation; GS-Rb1, ginsenoside Rb1; 5-HD, 5-hydroxydecanoate.

The level of LDH release is related to the damage to the H9c2 cells. The more severe the damage to cardiomyocytes, the higher the release of LDH. The amount of LDH released from the H9c2 cells subjected to HR was significantly higher than that released from the control cells (Figure 4, column B vs. A, ^*∗∗*^*p* < 0.01). The LDH released from the H9c2 cells subjected to HR after GS-Rb1 pretreatment was significantly lower than that from the HR group without GS-Rb1 (Figure 4, column C vs. B, ^*∗∗*^*p* < 0.01), and this protective effect was partially eliminated by the addition of 5-HD (Figure 4, column C vs. D, ^*∗*^*p* < 0.05). The release of LDH from H9c2 cells in the diazoxide the pretreatment group was significantly lower than that in the HR group (Figure 4, column E vs. B, ^*∗∗*^*p* < 0.01). Therefore, pretreatment with GS-Rb1 significantly reduced damage to H9c2 cells induced by HR, and this protective effect could be partially eliminated by 5-HD, while diazoxide produced a similar effect as GS-Rb1 during HR.

### 3.4. GS-Rb1 Stabilizes the Mitochondrial Membrane Potential (MMP) during HR

**Figure 5:**
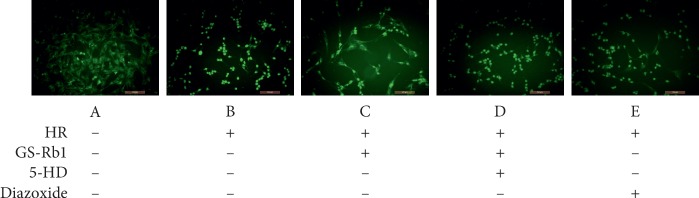
GS-Rb1 protects the mitochondrial membrane potential (MMP) of H9c2 cells during HR. The fluorescence intensity is negatively correlated with the MMP. (a) Control cells. (b) H9c2 cells showing high-intensity fluorescence during HR indicating reduced MMP. (c) H9c2 cells subjected to HR after pretreatment with GS-Rb1show significantly reduced fluorescence intensity compared with nontreated H9c2 cells. (d) H9c2 cells subjected to HR after simultaneous pretreatment with both GS-Rb1 and 5-HD show increased fluorescence intensity and cell rounding similar to nontreated H9c2 cells during HR. (e) H9c2 cells subjected to HR in the presence of diazoxide show reduced fluorescence similar to the protective effect seen with GS-Rb1. Cells were visualized under a fluorescent microscope (Olympus, Japan) (magnification 200×). HR, hypoxia-reoxygenation; GS-Rb1, ginsenoside Rb1; 5-HD, 5-hydroxydecanoate.

**Figure 6:**
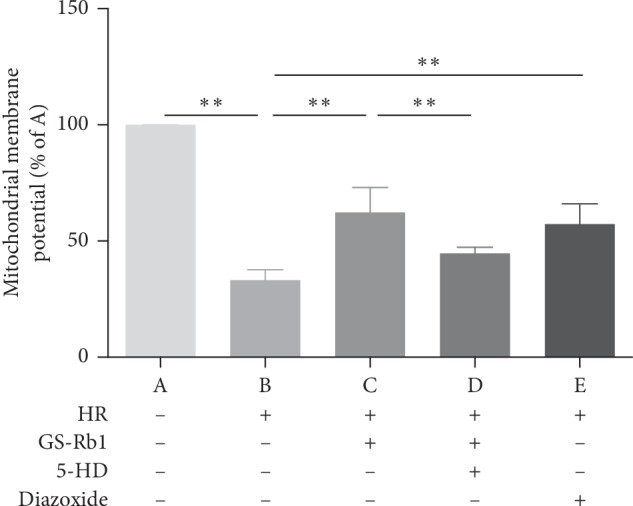
GS-Rb1 protects the mitochondrial membrane potential (MMP) of H9c2 cells subjected to HR. The MMP of the H9c2 cells during HR was significantly lower than that of the control cells (B vs. A ^*∗∗*^*p* < 0.01), and GS-Rb1 can significantly prevent the decrease of the MMP of H9c2 cells during HR (C vs. B ^*∗∗*^*p* < 0.01), similar to the effect of diazoxide (E vs. B ^*∗∗*^*p* < 0.01). The protective effect of GS-Rb1 on the MMP was prevented by co-pretreatment with 5-HD (D vs. C ^*∗∗*^*p* < 0.01; D vs. B *p* > 0.05). HR, hypoxia-reoxygenation; GS-Rb1, ginsenoside Rb1; 5-HD, 5-hydroxydecanoate.

The cell-permeable, green-fluorescent dye Rhodamine 123 (Rh123) was used to assess the MMP. The fluorescence microscopy photograph of the control H9c2 cells showed uniform low-intensity fluorescence, with normal cell morphology (Figure 5, panel A). Most of the H9c2 cells subjected to HR became round, showing high-intensity fluorescence (Figure 5, panel B) indicating perturbation of the MMP. The fluorescence intensity of both the GS-Rb1 and diazoxide pretreated cells subjected to HR was lower than that of cells subjected to HR without pretreatments and cells appeared more normal morphologically (Figure 5, panels C and E, respectively). Cells from the 5-HD and GS-Rb1 cotreatment group showed high-intensity fluorescence and rounding, similar to non-pretreated HR-subjected cells (Figure 5, panel D). Statistical analysis revealed that the MMP of the H9c2 cells during HR was significantly lower than that of the control group (Figure 6, column B vs. A, ^*∗∗*^*p* < 0.01), while the MMP of the HR-subjected H9c2 cells pretreated with GS-Rb1 or diazoxide was significantly higher than that of the HR group (Figure 6, column C vs. B, ^*∗∗*^*p* < 0.01; E vs. B, ^*∗∗*^*p* < 0.01, respectively), and the protective effect of GS-Rb1 could be eliminated by co-pretreatment with 5-HD (Figure 6, column D vs. C, ^*∗∗*^*p* < 0.01; column D vs. B, *p* > 0.05). The MMP assay showed that GS-Rb1 can effectively prevent the collapse of MMP during HR, similar to the action of diazoxide, and this protective effect could be eliminated by 5-HD.

### 3.5. GS-Rb1 Reduces the Formation of Reactive Oxygen Species (ROS) during HR

**Figure 7:**
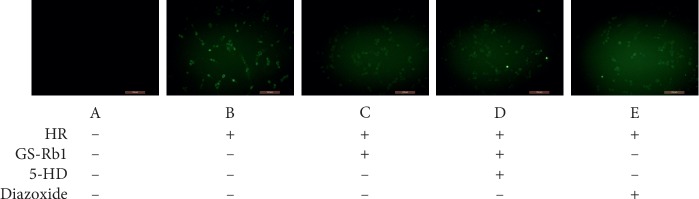
Effect of GS-Rb1 on the production of ROS in H9c2 cells. The fluorescence intensity was positively correlated with the amount of ROS produced in the cells. A, control cells. B, H9c2 cells showing higher intensity fluorescence during HR compared with control cells. C, GS-Rb1 can significantly reduce the fluorescence intensity of H9c2 cells during HR compared with nontreated cells. D, The effect of GS-Rb1 is eliminated by simultaneous pretreatment with 5-HD. E, diazoxide can significantly reduce the fluorescence intensity of H9c2 cells during HR, compared with nontreated cells. Cells were visualized by a fluorescent microscope (Olympus, Japan). Magnification: 200x. HR, hypoxia-reoxygenation; GS-Rb1, ginsenoside Rb1; 5-HD, 5-hydroxydecanoate.

**Figure 8:**
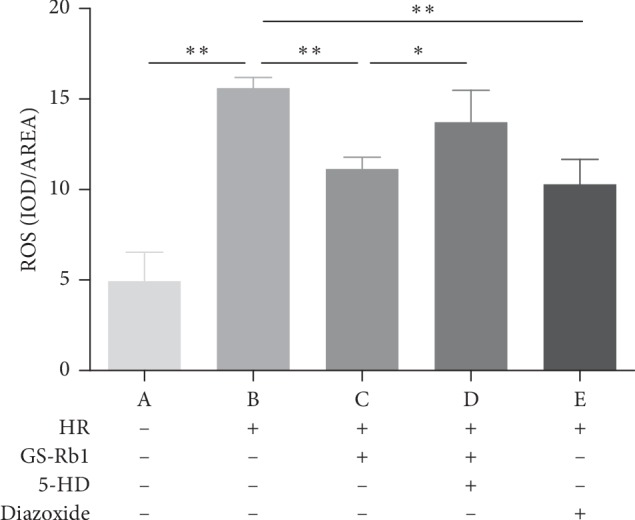
Effect of GS-Rb1 on the production of ROS in H9c2 cells subjected to HR. Column A, control H9c2 cells not subjected to HR. Column B, H9c2 cells subjected to HR without pretreatment showed increased ROS compared with nontreated cells. Column C, pretreatment with GS-Rb1 protected H9c2 cells from increased ROS during HR. Column D, simultaneous pretreatment with 5-HD prevented GS-Rb1 from protecting H9c2 cells against increased ROS during HR. Column E, pretreatment with diazoxide reduced the ROS of H9c2 cells during HR compared with nontreated cells. HR, hypoxia-reoxygenation; GS-Rb1, ginsenoside Rb1; 5-HD, 5-hydroxydecanoate.

The 2′-7′-dichlorodihydrofluorescein diacetate (DCFH-DA) assay was used to ROS. Fluorescence microscopy (Figure 7) showed that the fluorescence intensity of the normal cells was extremely low, while the green fluorescence of the H9c2 cells was obvious during HR. The fluorescence intensity of the H9c2 cells after pretreatment with GS-Rb1 or diazoxide was lower than that of the HR group. The fluorescence intensity of cell pretreated simultaneously with the 5-HD and GS-Rb1 was significantly higher than that of cells pretreated with GS-Rb1 alone. Statistical analysis (Figure 8) shows that the production of ROS in H9c2 cells during HR was significantly higher than that of the control (Figure 8, column B vs. A, ^*∗∗*^*p* < 0.01); however, after GS-Rb1 or diazoxide pretreatment, the amount of ROS produced by H9c2 cells was significantly lower than that of the HR group (Figure 8, column C vs. B, ^*∗∗*^*p* < 0.01; column E vs. B, ^*∗∗*^*p* < 0.01), and 5-HD eliminated the GS-Rb1 effects (Figure 8, column D vs. C, ^*∗*^*p* < 0.05; column D vs. B, *p* > 0.05). The ROS assay revealed that GS-Rb1 can effectively reduce the production of ROS during HR, and this effect could be eliminated by 5-HD.

### 3.6. GS-Rb1 Prevents the Continuous Opening of the Mitochondrial Permeability Transition Pore (mPTP) during HR

**Figure 9:**
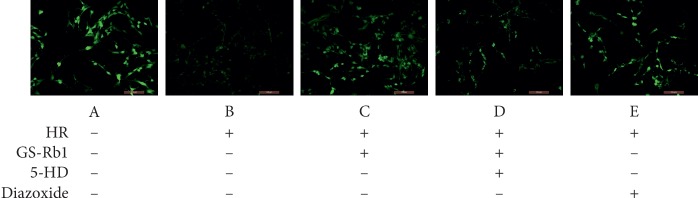
GS-Rb1 reduces the continuous opening of mPTP in H9c2 cells during HR. The fluorescence intensity is negatively correlated with the opening of mPTP. A, the control cells displayed uniform high-intensity fluorescence. B, the fluorescence intensity of the H9c2 cells during HR decreased significantly compared with control cells. C, pretreatment with GS-Rb1 significantly increases the fluorescence intensity of H9c2 cells during HR compared with nontreated cells. D The protective effect of GS-Rb1 can be partially eliminated by 5-HD. E, Pretreatment with diazoxide also significantly increases the fluorescence intensity of H9c2 cells during HR compared with nontreated cells. Cells were visualized by a fluorescent microscope (Olympus, Japan). Magnification: 200x. HR, hypoxia-reoxygenation; GS-Rb1, ginsenoside Rb1; 5-HD, 5-hydroxydecanoate.

**Figure 10:**
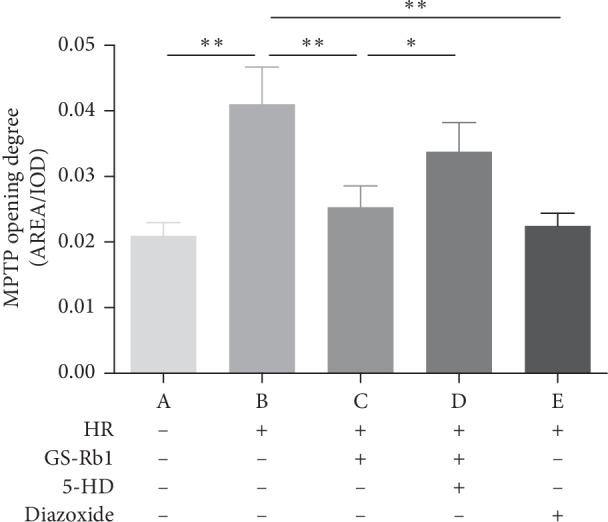
Effect of GS-Rb1 on the opening of mPTP in H9c2 cells. Column A, control H9c2 cells not subjected to HR. Column B, H9c2 cells subjected to HR without pretreatment showed increased mPTP opening compared with nontreated cells. Column C, pretreatment with GS-Rb1 protected H9c2 cells from mPTP opening during HR. Column D, simultaneous pretreatment with 5-HD prevented GS-Rb1 from protecting H9c2 cells against mPTP opening during HR. Column E, pretreatment with diazoxide reduced the mPTP opening of H9c2 cells during HR. HR, hypoxia-reoxygenation; GS-Rb1, ginsenoside Rb1; 5-HD, 5-hydroxydecanoate.

To assess the opening of the mPTP, we employed the acetomethoxy derivate of calcein (Calcein-AM) and CoCl_2_. Fluorescence microscopy showed that the control cells displayed uniform high-intensity fluorescence (Figure 9, panel A), while the fluorescence intensity of the H9c2 cells during HR decreased significantly compared with control cells (Figure 9, panel B). The fluorescence intensity of cells after pretreatment with GS-Rb1 or diazoxide was higher than that of nontreated cells, and the fluorescence of cells that was simultaneously pretreated with 5-HD, and GS-Rb1 was significantly reduced compared with that of cells pretreated with GS-Rb1 alone. Statistical analysis (Figure 10) shows that the continuous opening of mPTP in the H9c2 cells during HR was significantly greater than that of the control cells (Figure 10, column B vs. A, ^*∗∗*^*p* < 0.01) and the opening of mPTP in the H9c2 cells subjected to HR after pretreatment with GS-Rb1 or diazoxide was significantly lower than that in the cells subjected to HR without pretreatment (Figure 10, column C vs. B, ^*∗∗*^*p* < 0.01; column E vs. B, respectively, ^*∗∗*^*p* < 0.01), and treatment with 5-HD could partially eliminate the effect of GS-Rb1 (Figure 10, column D vs. C, ^*∗*^*p* < 0.05). The mPTP assay revealed that GS-Rb1 can effectively reduce the opening of mPTP during HR, and this effect could be partially eliminated by 5-HD.

### 3.7. GS-Rb1 Reduces the Apoptosis Rate of H9c2 Cells during HR

**Figure 11:**
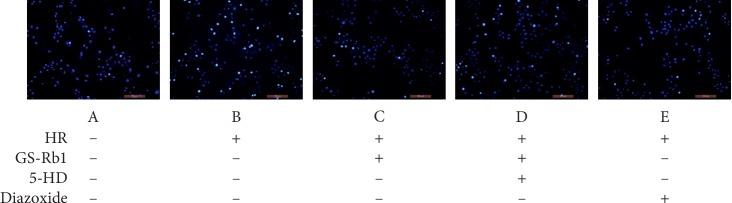
GS-Rb1 protects H9c2 cells from apoptosis during HR. The apoptosis rate was measured by Hoechst 33342 staining. GS-Rb1 significantly reduced the number of apoptotic nuclei of H9c2 cells during HR, similar to the effect of diazoxide, compared and this effect can be eliminated by 5-HD. Cells were visualized by a fluorescent microscope (Olympus, Japan) (magnification 200x). Apoptotic nuclei display high-intensity fluorescence. HR, hypoxia-reoxygenation; GS-Rb1, ginsenoside Rb1; 5-HD, 5-hydroxydecanoate.

**Figure 12:**
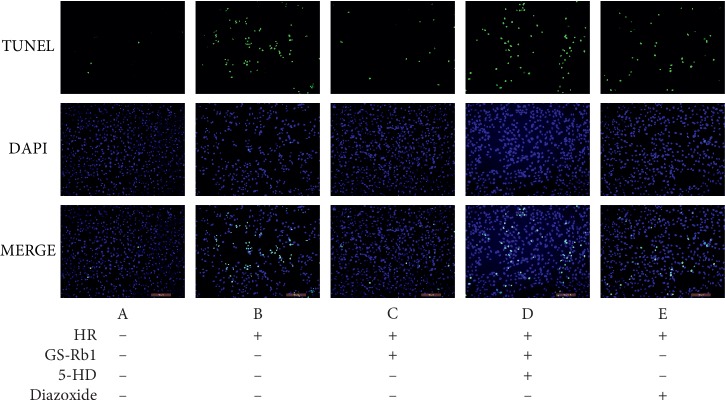
GS-Rb1 reduced the apoptosis rate of H9c2 cells during HR compared with nontreated cells. Apoptosis was measured using the TUNEL/DAPI assay. TUNEL staining revealed apoptotic nuclei, DAPI staining showed total nuclei, and MERGE visualizes the ratio of apoptotic nuclei to total nuclei (the apoptosis rate). Representative fluorescent images were obtained using a fluorescent microscope (Olympus, Japan) at a magnification of 200×. HR, hypoxia-reoxygenation; GS-Rb1, ginsenoside Rb1; 5-HD, 5-hydroxydecanoate.

**Figure 13:**
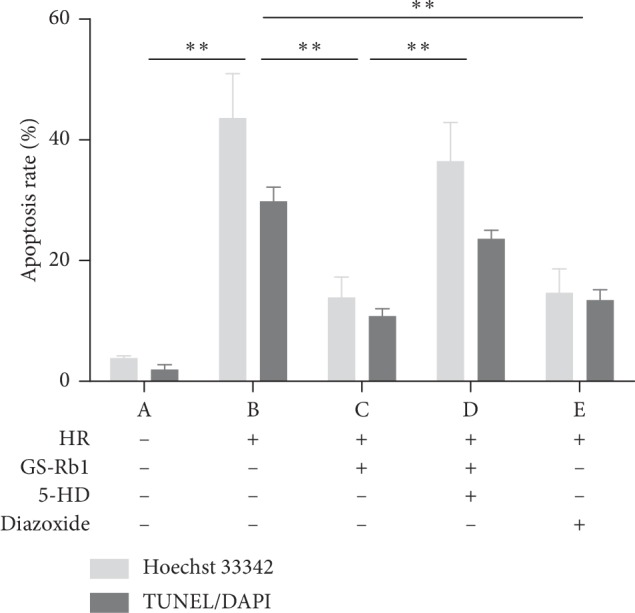
Protective effect of GS-Rb1 on the apoptosis rate of H9c2 cells during HR. Apoptosis was measured by Hoechst 33342 staining (light bars) or TUNEL/DAPI assay (dark bars). Column A, control H9c2 cells not subjected to HR. Column B, H9c2 cells subjected to HR without pretreatment showed increased apoptosis compared with nontreated cells. Column C, pretreatment with GS-Rb1 protected H9c2 cells from apoptosis during HR. Column D, simultaneous pretreatment with 5-HD prevented GS-Rb1 from protecting H9c2 cells during HR. Column E, pretreatment with diazoxide reduced the apoptosis rate of H9c2 cells during HR compared with nontreated cells. HR, hypoxia-reoxygenation; GS-Rb1, ginsenoside Rb1; 5-HD, 5-hydroxydecanoate.

The apoptosis rates measured by Hoechst 33342 staining (Figure 11) and TUNEL/DAPI staining (Figure 12) were approximately the same. Statistical analysis revealed that HR resulted in a significant increase in the apoptosis rate of H9c2 cells (Figure 13, column B vs. A, ^*∗∗*^*p* < 0.01). The apoptosis rate of H9c2 cells during HR after GS-Rb1 or diazoxide pretreatment was significantly decreased compared with nontreated cells subjected to HR (Figure 13, column C vs. B, ^*∗∗*^*p* < 0.01; Figure 13, column E vs. B, ^*∗∗*^*p* < 0.01, respectively). This protective effect of GS-Rb1 could be eliminated by coincubation with 5-HD (Figure 13, column D vs. C, ^*∗∗*^*p* < 0.01). Both methods showed that GS-Rb1 significantly reduced apoptosis induced by HR.

### 3.8. GS-Rb1 Reduces the Release of Cytochrome c and Reduces the Presence of Cleaved-Caspase-3 during HR

**Figure 14:**
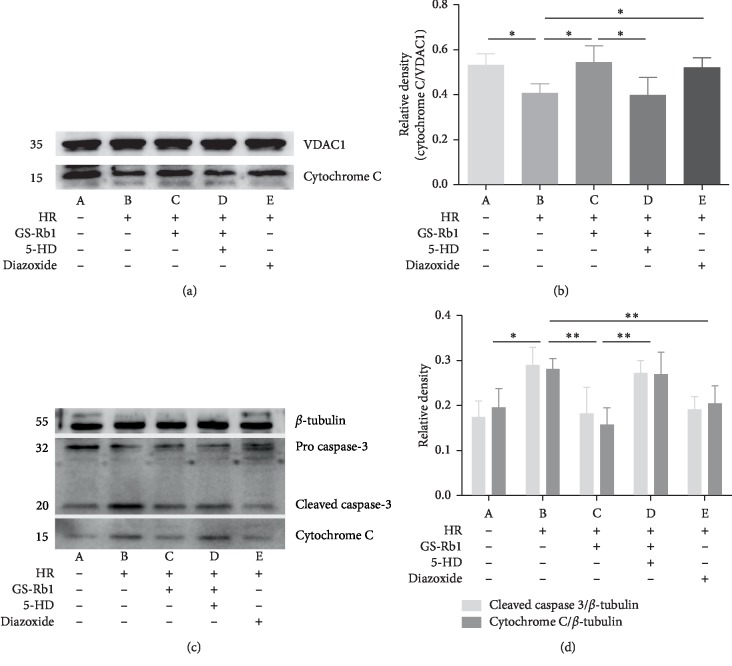
Expression of apoptosis-related proteins in H9c2 cells. (a) Representative western blot illustrating the mitochondrial localization of cytochrome c. (b) Densitometric analysis of the western blot of panel (a). (c) Representative western blot illustrating the cytoplasmic localization of cytochrome c and of cleaved-caspase-3. (d) Densitometric analysis the western blot of panel (c). VDAC1 and *β*-tubulin were loading controls. VDAC1, voltage-dependent anion-selective channel 1; HR, hypoxia-reoxygenation; GS-Rb1, ginsenoside Rb1; 5-HD, 5-hydroxydecanoate.

The presence of cytochrome c in the mitochondria is illustrated in Figure 14, panel (a) and the content of cytochrome c and cleaved-caspase-3 in the cytoplasm is illustrated in Figure 14, panel (c) as assayed by western blot. After optical density analysis, it was found that the cytochrome c content in mitochondria decreased during HR compared with control cells (Figure 14, panel (b), column B vs. A, ^*∗*^*p* < 0.05), and the release of cytochrome c into the cytoplasm increased (Figure 14, panel (d), column B vs. A, ^*∗*^*p* < 0.05). On the other hand, in GS-Rb1-pretreated or diazoxide-pretreated H9c2 cells subjected to HR, the amount of mitochondrial cytochrome c was increased, compared to untreated cells subjected to HR. (Figure 14 panel (b), column C vs. B and column E vs. B, respectively, ^*∗∗*^*p* < 0.01). At the same time, the release of cytochrome c into the cytoplasm was inhibited by GS-Rb1 and by diazoxide treatments (Figure 14 panel (d), column C vs. B and column E vs. B, respectively, ^*∗∗*^*p* < 0.01). The effect of GS-Rb1 could be eliminated by pretreatment with 5-HD (Figure 14 panel (b), column D vs. C, ^*∗*^*p* < 0.05 and Figure 14 panel (d), column D vs. C, ^*∗∗*^*p* < 0.01). The expression of cleaved-caspase-3 in the cytoplasm of each group was consistent with cytochrome c (Figure 14 panel (c) and Figure 14, panel (d)). The results of the western blot analysis showed that GS-Rb1 could effectively reduce the mitochondrial damage induced by HR in H9c2 cells, reduce the release of cytochrome c from mitochondria to the cytoplasm, and also reduce the expression of cleaved-caspase-3, inhibiting apoptosis induced by HR in H9c2 cells.

## 4. Discussion

mPTP is a nonselective high-conductance channel that spans the mitochondrial inner and outer membranes and is involved in regulating the mitochondrial Ca^2+^ balance, reducing free radical production and maintaining normal mitochondrial function. Under physiological conditions, mPTP is usually closed, and low-permeability mPTP makes the mitochondrial inner membrane selective, allowing only some metabolic substrates and ions to be exchanged [22, 23]. However, during MIRI, mitochondrial functional impairment leads to a decreased MMP, a reduced ATP synthesis, and an increased ROS concentration, resulting in an abnormal opening of mPTP. Continuous opening of mPTP causes mitochondrial permeability transition (MPT). MPT causes small molecules to enter the mitochondrial inner membrane, causing an increase in the osmotic pressure of the matrix, leading to mitochondrial edema. Because of the presence of the cristae structure, the mitochondrial inner membrane has a large surface area, allowing it to remain intact during matrix expansion; however, the edema of the matrix will decompose the outer membrane of the mitochondria due to its small surface area, resulting in the release of cytochrome c, apoptosis inducing factor (AIF), and other proapoptotic factors from the mitochondrial intramembranal space into the cytosol [24, 25]. Cytochrome c released into the cytosol binds to apoptotic protease activating factor-1 (Apaf-1) to form a complex which causes a cascade of caspase family proteins and cleaves pro-caspase-3 into cleaved-caspase-3. Cleaved-caspase-3 acts as an effector inducing the hydrolysis of different target proteins, ultimately leading to apoptosis [26].

Mitochondrial damage is a core process in the HR-induced apoptosis of H9c2 cells. MMP is decreased, and the ROS concentration is increased in H9c2 cardiomyocytes during HR. Decreased MMP causes a decrease in ATP synthesis, while excessive ROS promotes the oxidation of redox sites such as NADP, NADPH, and GSH on mPTP [27]. These reasons together promote the continuous opening of mPTP, which further aggravates the decline in MMP and further increases the volume of the matrix, and the ROS level continues to increase, forming a vicious cycle, eventually leading to destruction of the mitochondrial outer membrane, causing apoptosis.

This study revealed that diazoxide and GS-Rb1 can stabilize MMP and reduce ROS and ATP loss in H9c2 cardiomyocytes during HR, prevents the continuous opening of mPTP, and breaks the vicious cycle of continuous mitochondrial damage. In its closed state, mPTP maintains the low permeability of the mitochondrial inner membrane during HR, reduces the swelling of the mitochondrial matrix, and reduces the destruction of the outer membrane, thereby reducing the release of cytochrome C from the mitochondria to the cytosol. Ultimately, it caused a reduction of the apoptosis of H9c2 cardiomyocytes that occurred during HR.

The MitoK_ATP_ channel is an ATP-sensitive potassium channel present in the mitochondrial inner membrane. During MIRI, the opening of the MitoK_ATP_ channel may help control changes in mitochondrial matrix volume and help maintaining MMP production [28]. As a specific agonist of the MitoK_ATP_ channel, diazoxide may stabilize the mitochondrial matrix volume of H9c2 cardiomyocytes by activating the MitoK_ATP_ channel, thereby protecting the mitochondria. The mechanism by which GS-Rb1 protects mitochondria is unclear, but its effect can be partially offset by 5-HD, suggesting that the protective effect of GS-Rb1 on mitochondria may linked to the MitoK_ATP_ channel. However, since the effects of drugs on cells and mitochondria are very complex and an off-target effect of diazoxide and 5-HD exists [29], the exact nature of this link needs further research.

Dolowy [30] hypothesized that a calcium hydroxide buffer consisting of a few insoluble calcium phosphate minerals exists in the matrix that is the major calcium and pH buffer in mitochondria. The calcium hydroxide buffering capacity of the matrix is provided by calcium phosphates, and all of preconditioning methods, including potassium channel openers and calcium preconditioning, could increase this buffering capacity. Then, during hypoxia, calcium ions are expelled from the matrix, while hydroxyl ions increase its pH, which leads to the formation of the protonmotive force, ATP synthesis, and the control of the matrix volume, ultimately prolonging the mitochondrial survival rate during MIRI. This hypothesis may be conducive to explain how GS-Rb1 influences on mitochondria but still requires further testing.

This study investigated the protective effect of GS-Rb1 on H9c2 cardiomyocytes during HR. GS-Rb1 displays significant effects and benefits from low toxicity, so it can achieve a flexible balance in dose and efficacy, providing more choices as a potential drug for the treatment of MIRI.

## Data Availability

The data used to support the findings of this study are available from the corresponding author upon request.
